# A Severe Acute Respiratory Syndrome Coronavirus 2 Anti-Spike Immunoglobulin G Assay: A Robust Method for Evaluation of Vaccine Immunogenicity Using an Established Correlate of Protection

**DOI:** 10.3390/microorganisms11071789

**Published:** 2023-07-11

**Authors:** Mingzhu Zhu, Shane Cloney-Clark, Sheau-line Feng, Anand Parekh, Drew Gorinson, David Silva, Paul Skonieczny, Adjele Wilson, Raj Kalkeri, Wayne Woo, Miranda R. Cai, Louis Fries, Greg Glenn, Joyce S. Plested

**Affiliations:** 1 Clinical Immunology, Novavax, Inc., Gaithersburg, MD 20878, USA; 2 Biostatistics, Novavax, Inc., Gaithersburg, MD 20878, USA; 3 Clinical Development, Novavax, Inc., Gaithersburg, MD 20878, USA; 4 Discovery, Novavax, Inc., Gaithersburg, MD 20878, USA

**Keywords:** Binding antibodies, correlate of protection, IgG, ELISA, COVID-19, antibodies, Omicron BA.1/BA.5/XBB.1.5 SARS-CoV-2 variants, variants of concern

## Abstract

As the COVID-19 pandemic continues, variants of severe acute respiratory syndrome coronavirus 2 (SARS-CoV-2) continue to emerge. Immunogenicity evaluation of vaccines and identification of correlates of protection for vaccine effectiveness is critical to aid the development of vaccines against emerging variants. Anti-recombinant spike (rS) protein immunoglobulin G (IgG) quantitation in the systemic circulation (serum/plasma) is shown to correlate with vaccine efficacy. Thus, an enzyme-linked immunosorbent assay (ELISA)-based binding assay to detect SARS-CoV-2 (ancestral and variant strains) anti-rS IgG in human serum samples was developed and validated. This assay successfully met acceptance criteria for inter/intra-assay precision, specificity, selectivity, linearity, lower/upper limits of quantitation, matrix effects, and assay robustness. The analyte in serum was stable for up to 8 freeze/thaw cycles and 2 years in −80 °C storage. Similar results were observed for the Beta, Delta, and Omicron BA.1/BA.5/XBB.1.5 variant-adapted assays. Anti-rS IgG assay results correlated significantly with neutralization and receptor binding inhibition assays. In addition, usage of international reference standards allows data extrapolation to WHO international units (BAU/mL), facilitating comparison of results with other IgG assays. This anti-rS IgG assay is a robust, high-throughput method to evaluate binding IgG responses to S protein in serum, enabling rapid development of effective vaccines against emerging COVID-19 variants.

## 1. Introduction

The coronavirus disease 2019 (COVID-19) pandemic is caused by severe acute respiratory syndrome coronavirus 2 (SARS-CoV-2) [1]. The emergence of variants (such as Alpha, Beta, Gamma, Delta, and multiple Omicron subvariants) has led to ongoing transmission of the virus. Some SARS-CoV-2 variants (such as Omicron) have immune evasion properties, thus reducing the effectiveness of COVID-19 vaccines [2,3,4]. There is a need for additional correlates of protection (CoPs) for COVID vaccine efficacy to help track immune evasion and understand the needs of the vaccine development landscape (e.g., development of new vaccines based on variant S protein sequences) [5]. Validated CoPs help to extrapolate vaccine efficacy/immunogenicity results to populations or formulations/schedules not represented in clinical trials [6]. Of particular interest are CoPs for durability and level of vaccine-driven protection against ancestral and variant strains [6].

SARS-CoV-2 spike (S) protein on the viral surface is used to infect and enter target cells by binding to the receptor, human angiotensin-converting enzyme 2 (hACE_2_) [7]. S protein is thought to be a primary target of neutralizing/protective antibodies, including IgG [8]. Anti-S immunoglobulin G (IgG) is produced upon activation of the adaptive immune response, and a more robust IgG response has been seen in severe COVID-19 cases compared to mild cases [8]. Fong et al. have shown that anti-S IgG antibodies in the serum are a CoP for vaccine efficacy for ancestral strain when assessed for the COVID-19 vaccines NVX-CoV2373 and mRNA-1273 [6].

This article describes the development and validation of an in vitro bioanalytical IgG enzyme-linked immunosorbent assay (ELISA) to detect IgG antibodies against COVID-19 ancestral and variant (Beta, Delta, Omicron) strain rS protein, with a primary goal of quantitating vaccine responses and eventually establishing correlation of such responses with protection. The assay was assessed for precision, specificity, linearity, and other validation parameters, as well as correlation with pseudovirus neutralization, wild-type virus neutralization, and hACE_2_ binding inhibition assay results. Reference standard units (EU/mL) were converted to the WHO international unit in binding antibody units (BAU/mL) for the ancestral strain. The IgG assay described here is a robust, high-throughput, rapid option for detecting anti-rS IgG antibodies, which may be useful as a CoP for vaccine efficacy.

## 2. Materials and Methods

### 2.1. Assay Procedure

**Figure 1:**
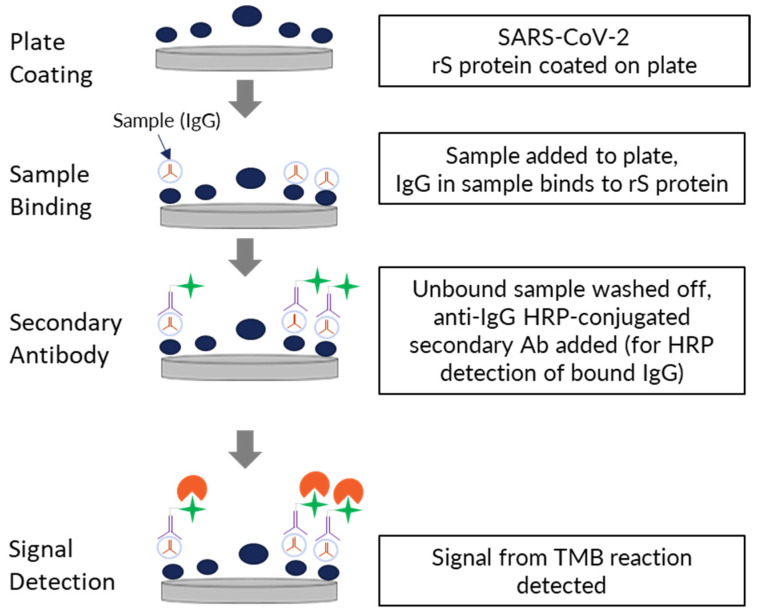
Procedure for performing the anti-rS IgG detection assay. Ab—antibody; IgG—immunoglobulin G; HRP—horseradish peroxidase; rS—recombinant spike; SARS-CoV-2—severe acute respiratory syndrome coronavirus 2; TMB—3,3′5,5′-tetramethylbenzidine.

An enzyme-linked immunosorbent assay (ELISA)-based assay was developed to detect IgG antibodies against SARS-CoV-2 S protein (full-length rS protein) from the ancestral Wuhan strain or variants (Beta, Delta, Omicron BA.1/BA.5/XBB.1.5). This 96-well format assay was designed for testing human sera from clinical trials of the NVX-CoV2373 vaccine (Figure 1).

Assay plates (Thermo Fisher Scientific, Waltham, MA, USA) were coated with 0.80 µg/mL SARS-CoV-2 rS protein (produced at Novavax, Inc., Gaithersburg, MD, USA) for 15 to 72 h at 2–8 °C. For assay development and initial validation, Novavax’s SARS-CoV-2 rS protein was prepared from the full-length (1273 amino acid), wild-type ancestral SARS-CoV-2 S protein based on Genbank gene sequence MN908947, nucleotides 21563-25384. As described by Tian et al. [9], a stable pre-fusion protein was produced by mutating the furin cleavage site and two residues in the CH domain. The recombinant protein was expressed in Sf9 insect cells and chromatographically purified to yield homotrimers displaying the N-terminal domain and receptor binding domain on the apical surface, as previously shown [9]. Coated plates were then washed with phosphate-buffered saline with Tween 20 (PBST) and blocked for 1 h with a blocking buffer (Thermo Fisher Scientific, Cat# 37542). Human serum samples (reference standards, quality controls, or test sera) were then added to the wells, allowing anti-rS protein IgG antibodies to bind (2 h of incubation). The plates were again washed with PBST, and then a goat anti-human IgG secondary antibody conjugated with horseradish peroxidase (HRP) (SouthernBiotech, Cat# 2040-05) was added and incubated for 1 h at room temperature. A final wash step was performed, followed by the addition of 3,3′5,5′-tetramethylbenzidine substrate (TMB, Sigma, Cat# T00440-1L). The reaction was stopped after 10 min by TMB stop solution (Scytek Laboratories, Cat# TSB999). The optical density (OD) of the chromogenic signal is directly proportional to the amount of anti-rS IgG captured on the plate, providing a quantifiable measurement of rS-specific IgG concentration in the serum sample.

### 2.2. Samples

Healthy human serum samples from before the COVID-19 pandemic (collected in 2016 through 2018) were obtained from BioIVT (Westbury, NY, USA) and Valley Biomedical (Winchester, VA, USA). COVID-19 convalescent serum was obtained from Sanguine BioSciences (Waltham, MA, USA) and BioIVT. Serum samples obtained from the Novavax clinical trial repository were from participants in phase 1 to 3 trials of the COVID-19 vaccine NVX-CoV2373 (Novavax, Gaithersburg, MD, USA).

Positive quality control (QC) samples (COVID-19 convalescent serum pools) known to have high or low anti-rS IgG levels were used. Negative controls (NC) were pre-COVID-19 sera negative for anti-rS IgG. QC samples were tested in duplicate wells on the first plate of each run.

For correlation analyses, serum samples were from the Novavax clinical trial 2019nCoV-311 (NCT05372588).

For analysis of conversion to WHO international units, the following samples were used: high QC (HQC)/low QC (LQC)/NC samples (as described above), an in-house reference standard COVID-19 convalescent serum pool, WHO international standard (NIBSC code 20/136) [10,11], and the WHO reference panel (NIBSC code 20/268). The reference panel contains 5 different pooled samples, ranging from high to low and negative antibody titers.

### 2.3. Validation Assays

#### 2.3.1. Precision

Twenty-seven samples were tested twice per assay run (in duplicate) in 6 different assay runs performed by 3 different analysts on 2 different days. The geometric mean concentration (GMC) was calculated for each sample from the 6 runs. Precision was then estimated by calculating the percent geometric coefficient of variation (%GCV), based on the variance component analysis using analyst and day as random effects and the samples as a fixed effect. The acceptance criteria for precision were that at least 80% of samples should have a %GCV ≤ 20%, while samples with IgG concentrations at or near the lower limit of quantitation (LLoQ) were permitted to have a %GCV ≤ 25%.

#### 2.3.2. Selectivity

Forty samples collected before the COVID-19 pandemic (which are thus expected to be negative for SARS-CoV-2-specific antibodies) were tested in the IgG assay. These samples were expected to have concentrations below LLoQ. Some of the samples had also been previously tested in an influenza hemagglutination inhibition assay (HAI) as previously described [12] to determine the effect of anti-influenza antibodies (HAI titers) on the IgG assay results.

#### 2.3.3. Specificity

To confirm the specific detection of anti-rS IgG, homologous antigen competition was assessed by incubating anti-rS IgG-positive samples with different amounts of SARS-CoV-2 rS for 1 h at room temperature before testing. Controls were samples incubated with assay buffer only. The acceptance criterion was that homologous protein incubation should reduce the detected IgG concentration by at least 50% in at least 80% of the samples tested.

To assess the potential for cross-reactivity with other betacoronavirus S proteins, samples were incubated with S protein from SARS-CoV-1 and MERS-CoV. Samples were also incubated with irrelevant proteins—respiratory syncytial virus fusion protein (RSV F) at 4 μg/mL and influenza virus hemagglutinin (HA) (A/Kansas/14/2017 virus-like particles [VLP]) at 4 HA units. These related and irrelevant viral proteins were, like SARS-CoV-2 rS, expressed in Sf9 insect cells and purified by generally parallel chromatographic methods. The targeted result was that incubation with irrelevant protein should not reduce the detected IgG concentration (≤20% reduction) in at least 80% of the samples tested.

Additionally, pre-COVID-19 samples from 5 participants vaccinated against RSV (Novavax clinical trial RSV-M-301, NCT02624947) and 5 participants vaccinated against influenza (Novavax clinical trial qNIV-E-301, NCT04120194) were tested in the anti-rS IgG assay (both pre- and post-vaccination samples). The targeted results were that vaccination against RSV or influenza using antigens produced in the same vaccine platform would not cause changes in detectable anti-rS IgG and that levels of anti-rS IgG should be low in the post-immunization samples despite vigorous responses to other highly immunogenic respiratory viral proteins.

For all specificity outcomes, the percent reduction in IgG concentration (% inhibition) was calculated as follows:

(1) $$\%\;Inhibition=[100-\frac{Results\;with\;protein\;incubation}{Results\;without\;protein\;incubation}\times 100]$$

#### 2.3.4. Matrix Effects

To assess the impact of hemolysis on the assay, 100% hemolyzed human blood (BioIVT BRH1369895) was spiked into 6 samples, and negative control to produce 50% hemolyzed or 25% hemolyzed samples (to represent severe hemolysis). After being tested in the IgG assay, percent recovery was calculated (result for the hemolyzed sample divided by the result from the non-hemolyzed sample). The acceptance criteria were a percent recovery between 80 and 120% of the reference value and that the NC should remain <LLoQ.

The same process was used to assess the impact of lipemia, with high lipemic serum with a high level of triglycerides (BioIVT BRH1119533, triglycerides 1473 mg/dL) being spiked into samples. The final triglyceride concentrations were 500 or 250 mg/dL (normal level, <150 mg/dL). The samples were then used in the IgG assay, and percent recovery was calculated. The acceptable range was recovery between 80 and 120% of the reference value, with the NCs below LLoQ.

#### 2.3.5. Linearity

Two phase 1 trial samples with high anti-rS IgG levels were tested in the assay undiluted and in a 1:2 dilution series (6 assay runs). Precision was calculated for each dilution point, and linear regression was performed for observed versus expected GMC. The expected EU/mL at each dilution was calculated from the overall GMC from all runs of the least diluted divided by the dilution factor for each dilution of each sample. The observed anti-rS protein IgG EU/mL at each dilution is the overall GMC at each dilution for each sample in all runs. To assess the ability of the assay to return values that accurately reflect the neat sample, the % relative bias at each dilution point was calculated as follows:

(2) $$\%\;Relative\;Bias=100\times \frac{\begin{array}{c} Observed\;overall\;anti\;rS\;\\ IgG\;GMC \end{array}-\begin{array}{c} Expected\;anti\;rS\;\\ IgG\;GMC \end{array}}{Expected\;anti-rS\;IgG\;GMC}$$

#### 2.3.6. Sensitivity

The lowest IgG level values that were accurately and precisely determined (as above) were assessed for the 2 linearity assessment samples in the linearity analysis. The LLoQ for the assay was set at 200 EU/mL during assay qualification and was confirmed during validation.

#### 2.3.7. Assay Robustness (Incubation Time and Plate Coating Time)

Incubation time robustness—The assay was conducted on 18 samples using lower and upper time limits for each incubation step as follows: plate coating time (15 and 72 h, lower and upper time limit, respectively), plate blocking time (60 min and 90 min), sample incubation on plate (110 min and 130 min), secondary antibody HRP on the plate (50 min and 70 min), TMB incubation on plate (8 min and 12 min). Assay results were then compared with the precision analysis runs performed under reference conditions. The acceptance criteria were that ≥80% of samples should have values within 80–120% of reference values. Percent recovery was calculated as follows:

(3) $$\%\;Recovery=100\times \frac{Test\;condition\;value}{Reference\;condition\;value}$$

Plate reading time robustness—The assay was conducted, and then plates were read for a colorimetric signal at three different time points (immediately, 15 min, or 30 min after stopping the TMB color development reaction). Assay results were then compared between the conditions to ensure percent differences were minimal.

#### 2.3.8. Sample Stability

Samples were stored at various temperatures: 6, 24, or 48 h at room temperature, 6 or 7 days at 2–8 °C, 29 days at –20 ± 10 °C, 6 or 24 months at –80 ± 10 °C. Samples were then tested in the assay, and the results were compared with the reference condition results from the precision analysis assay runs. Samples were also tested after 3, 6, 7, or 8 freeze/thaw cycles (1 h at room temperature followed by refreezing), and results were compared to the precision run results (only 1 freeze/thaw cycle). Percent recovery was calculated as shown above, with acceptable recovery between 80–120% of reference.

### 2.4. Variant Assays

The IgG assay was originally developed for the detection of IgG against ancestral strain S protein. However, with the emergence of variant strains, the assay was also adapted for Beta, Delta, Omicron BA.1, Omicron BA.5, and Omicron XBB.1.5 variants. The assay method was not altered; the sole difference in the assay was that the ancestral S protein used to coat the plate was replaced by the relevant variant sequence protein. A similar validation process as described above was followed.

### 2.5. Correlation Analyses

Each sample was assessed in the anti-rS IgG assay described here. The same samples were also tested in wild-type virus neutralization [13], pseudovirus neutralization [14], and hACE_2_ binding inhibition assays [15]. Linear regression analysis was conducted using GraphPad Prism software (San Diego, CA, USA; Version 9.3.1) to determine the correlation between anti-rS IgG results and other assay results. 

### 2.6. Conversion to WHO International Units

The WHO international standard was used to quantitate anti-rS antibodies as binding antibody units per mL (BAU/mL). As the assay described here is reported in ELISA units per mL (EU/mL), calibration was performed to convert these results to BAU/mL. High QC/low QC/NC samples (as described above), an in-house reference standard COVID-19 convalescent serum pool, WHO international standard (NIBSC code 20/136), and the WHO reference panel (NIBSC code 20/268 [10,11]) were diluted and tested in the assay. When the in-house reference was used, the results were reported in EU/mL, and when the WHO international standard was used for the reference curve, the results were reported in BAU/mL. The conversion factor between EU/mL and BAU/mL was then determined.

## 3. Results

### 3.1. Ancestral Strain Assay Validation Parameters

Inter-assay, intra-assay, and total precision were <20% GCV for all 27 serum samples representing all concentration ranges (low, medium, and high concentrations) (Table 1). This included the sample with the highest IgG level available (202,618 EU/mL), covering a range of 3.86 orders of magnitude.

Assay selectivity met acceptance criteria of ≥80% of samples having IgG levels below LLoQ. Of 40 serum samples collected before the COVID-19 pandemic (collected in 2016 through 2018), 35 had anti-rS IgG levels below LLoQ of 200 EU/mL (Table S1). Some of these samples were known to have high influenza hemagglutination inhibition assay titers (data not shown). Thus, the IgG assay is not affected by the presence of antibodies to other common respiratory pathogens.

Assay specificity also met the acceptance criteria. When 6 samples (from a phase 1 trial) were incubated with SARS-CoV-2 rS protein, IgG detection was strongly inhibited by >50% for all 6 samples, indicating that the assay is specific to SARS-CoV-2 rS protein (Table 2). When the same 6 samples were incubated with rS proteins of MERS-CoV and SARS-CoV-1, the phase 1 trial sera showed much less inhibition of the IgG signal, suggesting little cross-reactivity with MERS-CoV and SARS-CoV-1 in the assay. We did not test cross-reactivity with HCoV-OC34 or HKU1 sequence spike proteins, but these proteins have significantly lower sequence homology with SARS-CoV-2 spike than SARS-CoV-1 and MERS-CoV and have been shown to produce only minimal levels of cross-reactivity (16). Here, the absence of signals significantly > LLoQ in pre-pandemic sera, despite the widespread seropositivity to the endemic seasonal coronaviruses, is consistent with minimal cross-reaction.

The same 6 samples were also incubated with irrelevant proteins produced on the same vaccine platform (RSV F protein or influenza virus-like particles [VLP]) (Table 2). Among clinical trial samples, there was no significant inhibition (defined as >20%) of IgG detection by irrelevant proteins for 5 of 6 (83.6%) samples for RSV incubation and for 6 of 6 (100%) samples for influenza VLP.

Additionally, among 5 participants vaccinated against RSV and 5 vaccinated against influenza, anti-rS IgG levels (using immunogens manufactured on the same insect cell platform) did not change in post-vaccination versus pre-vaccination samples (Table S2). One RSV-vaccinated individual showed a 2.26-fold increase in anti-rS IgG concurrent with immunization, but the increase was from a <LLoQ value to a value slightly above LLoQ.

Free hemoglobin and lipemic matrix had minimal impact on the assay (Table 3).

**Figure 2:**
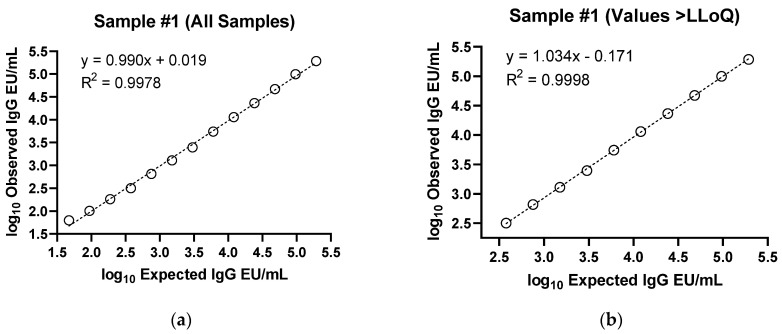

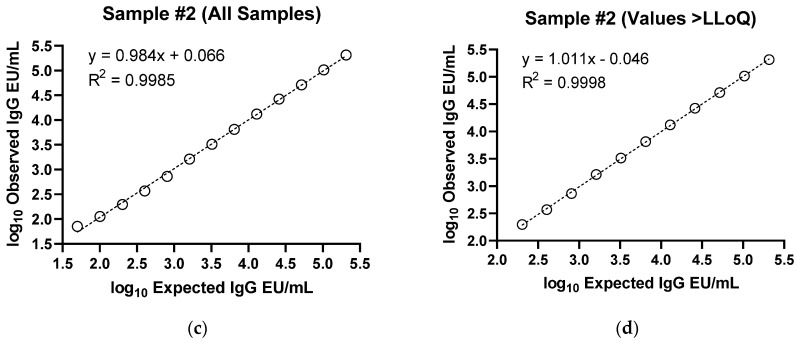
IgG assay ancestral strain linearity. Two samples positive for anti-rS IgG were diluted in 1:2 series and tested in 6 assay runs. Linearity was then evaluated by calculating the precision and accuracy of the concentration at each dilution and the slope of linear regression lines for each sample. For both samples, dilutions are shown from undiluted to 1:512 dilution. (**a**) Sample #1 with all samples shown, (**b**) Sample #1 with only values above LLoQ (200 EU/mL), (**c**) Sample #2 with all samples shown, and (**d**) Sample #2 with only values above LLoQ are presented.

Linearity of the assay was demonstrated, with R^2^ values of 0.9998 for the 2 tested samples (Figure 2 and Table 4). LLoQ was assigned at 200 EU/mL, based on the lowest concentration values that could be detected accurately and precisely in these two samples (and precision data [Table 1] on samples with anti-rS levels approximating 200 EU/mL supported this selection). Based on phase 1 samples, the upper limit of quantitation (ULoQ) was provisionally assigned as at least 206,767 EU/mL. However, based on 5 serum samples from phase 2 and phase 3 trials of the COVID vaccine NVX-CoV2373, the ULoQ is currently estimated to be at least 2,904,275 EU/mL.

The robustness of the assay was demonstrated for incubation time (Tables S3 and S4). At the lower incubation time, 18 of 18 (100%) samples had recovery within the acceptable range (80–120% of reference). At the upper incubation time, 16 of 18 (88.9%) samples had recovery within the acceptable range.

The robustness of the assay was also demonstrated for plate reading time (Table S5). All three QC samples showed differences ≤2.1% when the plates were read within 30 min of color development.

**Figure 3:**
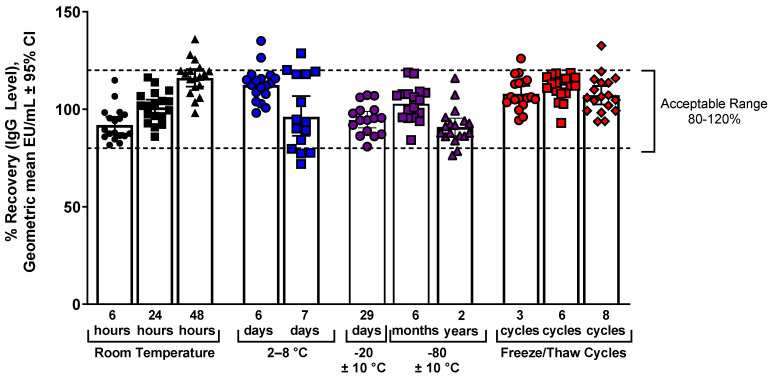
IgG assay ancestral strain temperature and freeze/thaw stability. Samples were stored at different temperature conditions or were subjected to multiple freeze/thaw cycles and then used in the IgG assay. The acceptable recovery range was 80% to 120% of the reference condition values (dashed lines).

The stability of samples was tested at different storage temperatures (room temperature, refrigeration [2–8 °C], and freezing [−20 °C or −80 °C]). Samples were also assessed after multiple freeze/thaw cycles (Figure 3). The samples were stable up to 8 freeze/thaw cycles and up to 2 years in −80 °C freezer storage, as shown by recovery within the acceptable recovery range of 80–120% of reference.

### 3.2. Variant Strain Assay Validation

**Figure 4:**
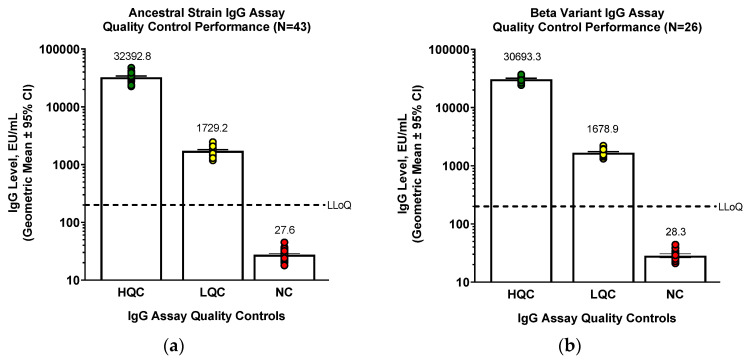

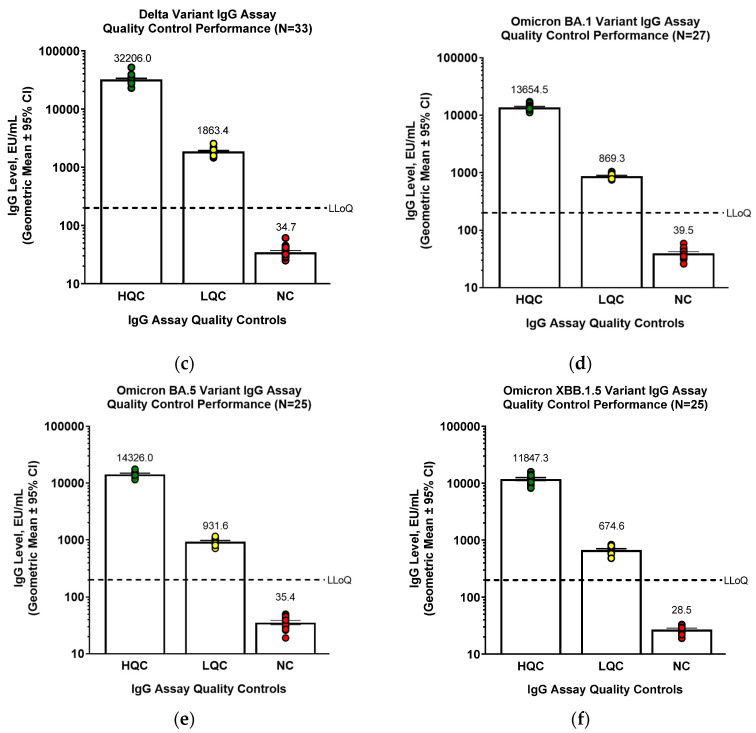
IgG assay ancestral strain and variants quality control standards performance. (**a**) For ancestral strain, (**b**) Beta variant, (**c**) Delta variant, (**d**) Omicron BA.1 variant, (**e**) Omicron BA.5 variant, or (**f**) Omicron XBB.1.5 variant, QC samples were tested in the IgG detection assay to examine assay performance. One value was identified as an outlier for the NC group of XBB.1.5 and was removed from the analysis.

Although the original assay was developed using ancestral strain rS protein, the assay was used for variant strains as they emerged, including Beta, Delta, Omicron BA.1, Omicron BA.5, and Omicron XBB.1.5 variants. Quality control samples performed similarly well for variants compared to ancestral strain (Figure 4). Pooled convalescent serum known to have high (HQC) or low (LQC) levels of anti-rS IgG or known to be negative for anti-rS IgG (NC) was used for the assessment.

Results for the assay validation parameters for the Beta, Delta, and Omicron BA.1/BA.5/XBB.1.5 variants were similar to those for the ancestral strain (Table S6). For the Beta variant, inter-assay, intra-assay, and total precision had <20% GCV for 18 of 20 (90%) samples. Selectivity was demonstrated, as 36 of 40 (90.0%) pre-COVID-19 samples showed <LLoQ results in the assay. Specificity was also demonstrated, with 8 of 8 samples showing >50% reduction in IgG detection when incubated with homologous S protein. SARS-CoV-1 and MERS-CoV S protein reduced IgG detection in 5 of 8 and in 3 of 8 samples, respectively. RSV F protein reduced IgG detection in 3 of 8 samples; influenza hemagglutinin did not reduce IgG detection in any samples. Among 5 participants vaccinated against RSV and 5 vaccinated against influenza, none of the post-vaccination samples showed changes in anti-rS IgG detection compared with pre-vaccination. Linearity was successfully demonstrated (R^2^ = 0.9999 and 0.9996), with LLoQ defined as 200 EU/mL and ULoQ determined to be at least 490,731 EU/mL.

For the Delta variant, 19 of 20 (95%) samples had inter-assay, intra-assay, and total precision % GCV < 20%. Of 40 pre-COVID-19 serum samples, 33 (82.5%) showed <LLoQ results in the assay. Of 7 samples, 6, 3, and 7 samples showed >50% reduction in IgG detection when incubated with homologous S proteins (ancestral, Beta, and Delta, respectively). SARS-CoV-1 and MERS-CoV S protein reduced IgG detection in 2 of 7 and in 1 of 8 samples, respectively. RSV F protein did not reduce IgG detection in any samples; influenza hemagglutinin reduced IgG detection in 1 of 7 samples. Among 5 participants vaccinated against influenza, none of the post-vaccination samples had changes in anti-rS IgG compared with pre-vaccination. Of the 5 participants vaccinated against RSV, only 1 showed changes in anti-rS IgG levels. Linearity was successfully demonstrated (R^2^ = 0.9999 and 0.9998), LLoQ was defined as 200 EU/mL, and ULoQ was determined to be at least 501,789 EU/mL.

For Omicron BA.1, inter-assay, intra-assay, and total precision had < 20% GCV for 20 of 21 (95.2%) samples. Of 40 pre-COVID-19 serum samples, 31 (77.5%) showed <LLoQ results. Of 8 samples, 7 to 8 samples showed >50% reduction in IgG detection when incubated with homologous S protein. RSV F protein did not reduce IgG detection in 6 of 8 (75%) samples. Influenza hemagglutinin did not reduce IgG detection in 7 of 8 (87.5%) samples. Among 5 participants vaccinated against influenza, none of the post-vaccination samples had changes in anti-rS IgG compared with pre-vaccination. Of the 5 participants vaccinated against RSV, only 1 showed changes in anti-rS IgG levels. Linearity was successfully demonstrated (R^2^ = 0.986 and 0.966), LLoQ was defined as 200 EU/mL, and ULoQ was determined to be at least 391,124 EU/mL. Robustness for plate coating time was demonstrated, as 17 of 21 samples (81.0%) had recovery within 80–120% of the baseline values.

For Omicron BA.5, all 21 samples had inter-assay, intra-assay, and total precision % GCV < 20%. Of 40 pre-COVID-19 serum samples, 34 (85%) showed <LLoQ results. Of 8 samples, 6 to 8 samples showed >50% reduction in IgG detection when incubated with SARS-CoV-2 S protein (7/8 for Omicron BA.5, 6–7/8 for Omicron BA.1, 6/8 for ancestral strain). RSV F protein did not reduce IgG detection in 7 of 8 (87.5%) samples; Ebola glycoprotein did not reduce IgG detection in any samples. Linearity was successfully demonstrated (R^2^ = 0.9988 and 0.9991). LLoQ was defined as 200 EU/mL, and ULoQ was determined to be at least 990,591 EU/mL. Robustness for plate coating time was demonstrated, as 17 of the 21 samples (81.0%) were within 80–120% of the baseline values.

For Omicron XBB.1.5, all 21 serum samples had inter-assay, intra-assay, and total precision % GCV < 20%. Of 40 pre-COVID-19 samples, 33 (82.5%) showed <LLoQ results. Of 8 samples tested, all samples showed a >50% reduction in IgG detection when incubated with SARS-CoV-2 S protein (ancestral strain, Omicron XBB.1.5, or Omicron BA.5). Incubation with RSV F protein did not reduce IgG detection in 7 of 8 (87.5%) samples, and Ebola glycoprotein incubation did not reduce IgG detection in any of the 8 samples. Linearity was successfully demonstrated (R^2^ = 0.9985 and 0.9991). LLoQ was defined as 200 EU/mL, and ULoQ was determined to be at least 682,680 EU/mL. Robustness for plate coating time was demonstrated, as all 21 tested samples had recovery within 80–120% of the baseline values.

### 3.3. Assay Correlation with Other Markers

Based on the presence of anti-N antibodies (Roche Cobas Elecsys Assay, University of Washington), serum samples were classified as either seropositive (with prior exposure to natural SARS-CoV-2 infection) or seronegative (naïve subjects). Natural SARS-CoV-2 infection also induces anti-rS IgG as part of the body’s immune response. Levels of anti-rS IgG in the serum from both seropositive and seronegative subjects were evaluated using the anti-rS IgG assays for ancestral, Omicron BA.1, and Omicron BA.5 strains, followed by correlation with the neutralizing antibody levels (either live virus-based microneutralization assays or pseudovirus-based neutralization assays) and the hACE_2_ binding inhibition assay [15].

**Figure 5:**
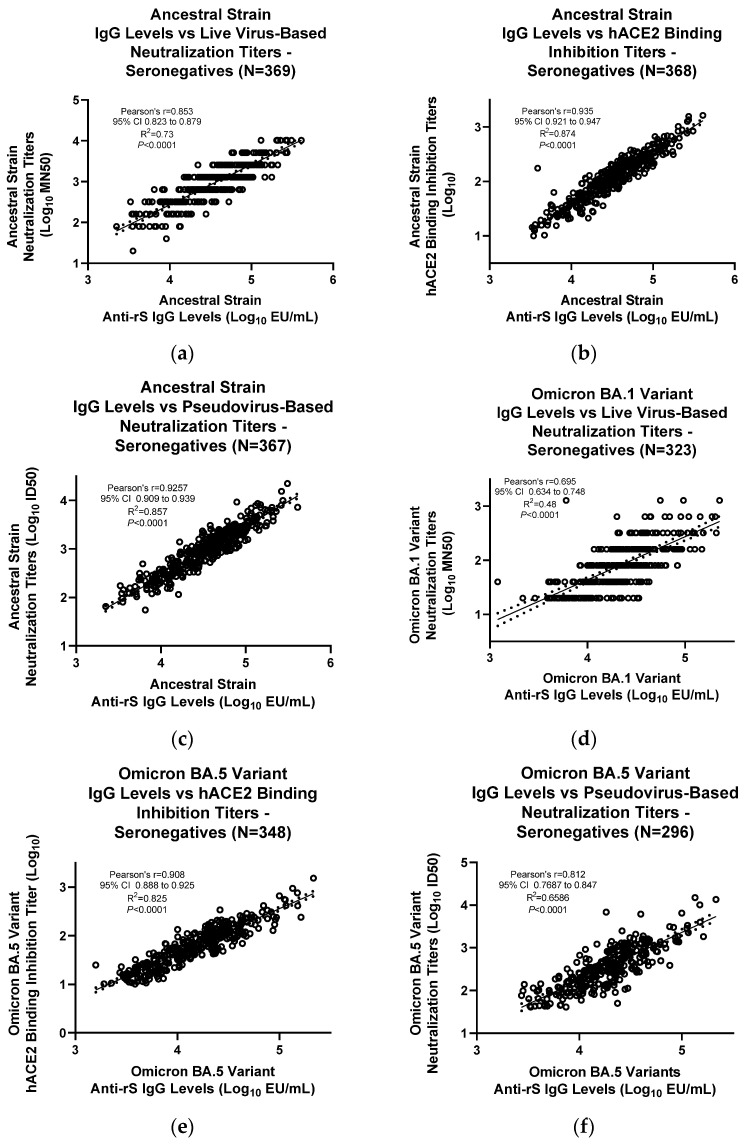
Correlation of IgG assay results with other immunogenicity assays in SARS-CoV-2 seronegative serum samples. Results from the IgG assay analyses were correlated with results from other assays for (**a**–**c**) ancestral strain, (**d**) Omicron BA.1 variant, or (**e**,**f**) Omicron BA.5 variant. For (**c**,**f**), a pseudovirus neutralization assay was performed as previously described [14]. For (**a**,**d**), a live wild-type virus neutralization assay was performed as previously described [13]. For (**b**,**e**), an hACE_2_ binding inhibition assay was performed as previously described [15]. Linear regression analysis was performed to compare results from the IgG assay and the other assays. ID50—median infectious dose; LOD—limit of detection; Log^10^—logarithm with base 10; MN50—50% microneutralization.

For seronegative samples, the IgG assay ancestral strain results correlated significantly with results from the live wild-type virus neutralization assay (R^2^ = 0.73, Pearson’s r = 0.853, *p* < 0.0001) (Figure 5a), hACE_2_ binding inhibition assay (R^2^ = 0.874, Pearson’s r = 0.935, *p* < 0.0001) (Figure 5b), and pseudovirus-based neutralization assay (R^2^ = 0.857, Pearson’s r = 0.926, *p* < 0.0001) (Figure 5c). IgG assay Omicron BA.1 results correlated significantly with results from the live wild-type virus neutralization assay (R^2^ = 0.48, Pearson’s r = 0.695, *p* < 0.0001) (Figure 5d). IgG assay Omicron BA5 results correlated significantly with results from the hACE_2_ binding inhibition assay (R^2^ = 0.825, Pearson’s r = 0.906, *p* < 0.0001) (Figure 5e) and pseudovirus-based neutralization assay (R^2^ = 0.659, Pearson’s r = 0.812, *p* < 0.0001) (Figure 5f).

**Figure 6:**
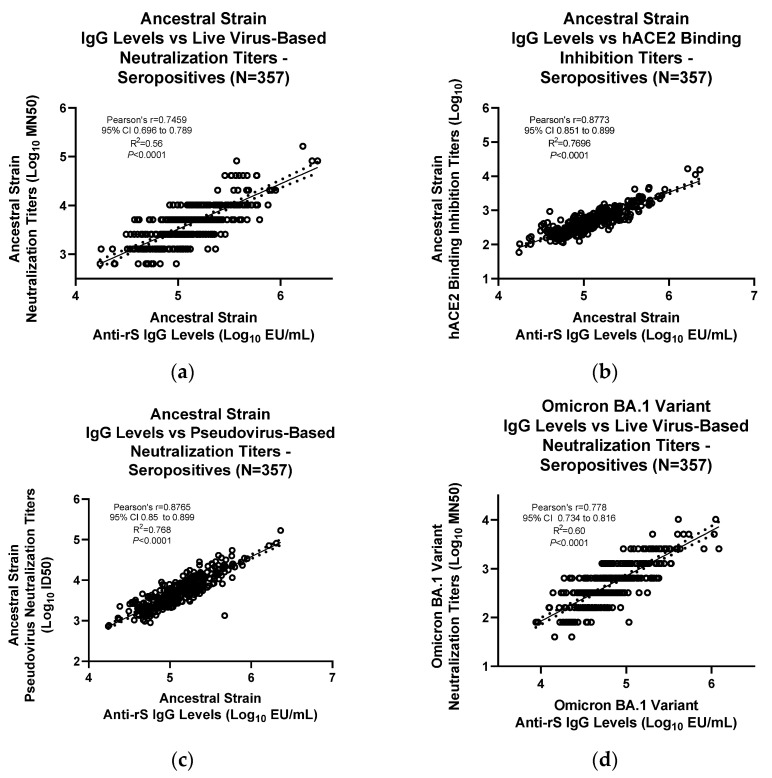

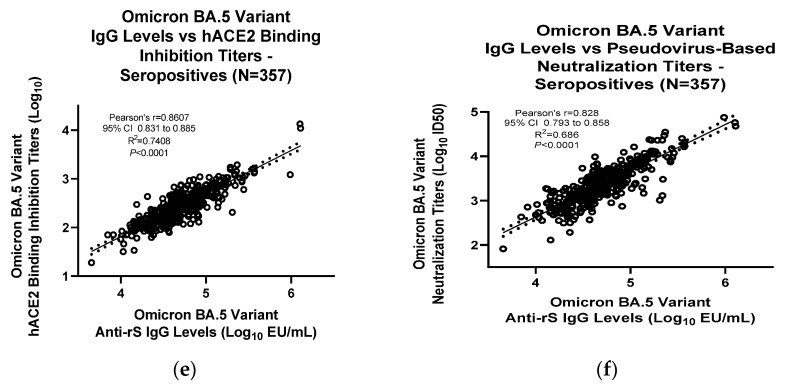
Correlation of IgG assay results with other immunogenicity assays in SARS-CoV-2 seropositive serum samples. Results from the IgG assay analyses were correlated with results from other assays for (**a**–**c**) ancestral strain, (**d**) Omicron BA.1 variant, or (**e**,**f**) Omicron BA.5 variant. For (**c**,**f**), a pseudovirus neutralization assay was performed as previously described [14]. For (**a**,**d**), a live wild-type virus neutralization assay was performed as previously described [13]. For (**b**,**e**), an hACE_2_ binding inhibition assay was performed as previously described [15]. Linear regression analysis was performed to compare results from the IgG assay and the other assays. ID50—median infectious dose; LOD—limit of detection; Log_10_—logarithm with base 10; MN50—50% microneutralization.

Similarly, for seropositive samples, the IgG assay ancestral strain results correlated significantly with results from the live wild-type virus neutralization assay (R^2^ = 0.56, Pearson’s r = 0.746, *p* < 0.0001) (Figure 6a), hACE_2_ binding inhibition assay (R^2^ = 0.769, Pearson’s r = 0.877, *p* < 0.0001) (Figure 6b), and pseudovirus-based neutralization assay (R^2^ = 0.768, Pearson’s r = 0.876, *p* < 0.0001) (Figure 6c). IgG assay Omicron BA.1 results correlated significantly with results from the live wild-type virus neutralization assay (R^2^ = 0.60, Pearson’s r = 0.778, *p* < 0.0001) (Figure 6d). IgG assay Omicron BA5 results correlated significantly with results from the hACE_2_ binding inhibition assay (R^2^ = 0.741, Pearson’s r = 0.861, *p* < 0.0001) (Figure 6e) and pseudovirus-based neutralization assay (R^2^ = 0.686, Pearson’s r = 0.828, *p* < 0.0001) (Figure 6f).

### 3.4. Conversion to WHO International Standard Units (BAU/mL)

To convert between the assay ELISA unit (EU/mL) and the WHO international unit (BAU/mL) [10,11], assay calibration was performed. The calibration runs, and analysis showed that the conversion factor to convert EU/mL to BAU/mL was 22, so an IgG level in EU/mL can be expressed in BAU/mL after being divided by 22 (Table S7).

## 4. Discussion

This article details the validation of an ELISA-based anti-rS IgG assay, which will be used for evaluating COVID-19 vaccine immunogenicity and may provide a useful roadmap for the establishment and validation of an IgG assay for vaccines against other pathogens. The original assay developed for ancestral strain SARS-CoV-2 showed acceptable inter- and intra-assay precision, specificity, selectivity, linearity, lower/upper limits of quantitation, matrix interference effects, and assay robustness (incubation/plate reading time) parameters. Serum samples were stable for up to 8 freeze/thaw cycles and up to 2 years in −80 °C freezer storage. The IgG assay results correlated significantly with three different functional assays, including live wild-type virus and pseudovirus-based neutralization assays and an hACE_2_ binding inhibition assay (all strongly positively correlated with *p* values < 0.001). In addition, our ELISA units (EU/mL) can be converted to WHO international units (BAU/mL), allowing for comparison of results with other IgG assays.

Of 40 samples collected prior to the COVID-19 pandemic, 35 had anti-rS IgG levels below LLoQ, as expected. Five of the samples showed very low anti-SARS-CoV-2 spike IgG levels (215–422 EU/mL) that were slightly above the assay LLoQ. Antibodies to human seasonal betacoronaviruses could contribute to this low occurrence and low-level background reactivity since there is approximately 30% sequence homology between SARS-CoV-2 and seasonal betacoronaviruses spike sequences [16], and antibodies to the latter are near ubiquitous. Nonetheless, in the context of either SARS-CoV-2 infection or immunization, these signals are trivial relative to the foreground.

Anti-rS IgG levels in the serum of either seropositive or seronegative subjects correlated well with the other measures of immunogenicity, including live wild-type virus/pseudovirus-based neutralization assays and hACE_2_ binding inhibition assays for the ancestral and variant strains tested to date [15]. Based on these results, the validated IgG assay described here could be useful for measuring the immunogenicity of vaccines against both ancestral and emerging variants. Evaluation of the same sera for different variants in both live wild-type and pseudovirus-based neutralization assays showed different proportions of neutralizing antibody levels (e.g., for Omicron variants, a booster was needed to increase the neutralization titers). This suggests that the strength of the correlation of IgG levels to other neutralization assays might vary for different variant strains and needs to be evaluated and interpreted carefully. Future work will focus on developing this IgG assay for variants as they emerge. As the validated IgG assay results can be expressed in international units (BAU), vaccine correlates of protection levels to ancestral strain SARS-CoV-2 can be determined, as demonstrated in the literature [6].

The utility of this validated anti-rS IgG assay is that it measures all binding antibodies, including non-neutralizing antibodies, in clinical samples; it cannot directly distinguish neutralizing antibodies from binding antibodies. It is important to note that the results from this assay correlate significantly with other immunogenicity measures (including neutralization assays) for all the strains tested to date. However, for the emerging variant strains, it might also be worth considering the neutralizing antibody levels as a CoP for vaccine efficacy, which is an ongoing effort in the COVID-19 vaccine research. When the anti-rS IgG assay was adapted for the Beta, Delta, and Omicron BA.1/BA.5/XBB.1.5 variants, the variant-adapted assays showed similar results for validation parameters. This suggests that the validated anti-rS IgG assay framework established here in this work will also be useful for emerging SARS-CoV-2 variants. To the extent that neutralizing antibodies are accepted as a CoP for variants, variant anti-rS IgG is also highly likely to be a valuable CoP because it is uniformly, strongly, and positively correlated with neutralizing antibodies across all variants to date. This validated assay could play a pivotal role in evaluating the immune response in clinical studies, as bivalent or variant-adapted vaccines are developed to prevent COVID-19. To our knowledge, this is the first validation report of an anti-S IgG ELISA assay for SARS-CoV-2 XBB.1.5 strain. This is important as the majority of current COVID-19 infections are caused by XBB.1.5 strain, and our report paves the way for testing XBB.1.5 anti-S IgG in clinical samples.

## 5. Conclusions

The IgG assay is precise, robust, linear, and specific for SARS-CoV-2 S protein and is useful for both ancestral strain and variants (tested for Beta, Delta, and Omicron BA.1/BA.5/XBB.1.5). Results from the IgG assay correlated significantly with pseudovirus/live wild-type virus neutralization and hACE_2_ binding inhibition assays for all variants tested, ranging from the ancestral virus to XBB.1.5. Results in EU/mL can be converted to BAU/mL, making it possible to compare results from this assay with results from other IgG assays performed in different laboratories. The IgG assay described here will be useful for evaluating vaccine immunogenicity and also possibly as a correlate of the protection analysis method for vaccine efficacy. Future work will focus on modifying the assay for emerging variants, including BQ.1.1. XBB.1.16, and others as needed.

## Figures and Tables

**Table 1:** IgG assay ancestral strain precision (intra-assay and inter-assay precision).

Sample ^1^	Anti-rS Protein Ab GMC (EU/mL)	Inter-Assay %GCV	Intra-Assay %GCV	Total %GCV
Overall ^2^	N/A	0.9	13.5	13.5
1	47,648	0	13.7	13.7
2	16,537	5.8	13.6	14.9
3	234	6.7	11.0	12.9
4	9303	7.2	17.2	18.7
5	180,688	0	7.8	7.8
6	202,618	6.4	8.8	10.9
7	56,315	2.5	7.7	8.1
8	2146	0	14.2	14.2
9	4890	0	14.1	14.1
10	1447	0	14.0	14.0
11	105,191	0	9.6	9.6
12	510	0	14.6	14.6
13	1067	3.3	12.3	12.7
14	187	1.9	9.4	9.6
15	4629	0	12.8	12.8
16	824	0	14.5	14.5
17	35,042	0	10.8	10.8
18	74,679	0	9.6	9.6
19	106,859	10.9	14.1	17.8
20	7075	0	13.5	13.5
21	101,122	0	16.6	16.6
22	26,750	9.4	10.6	14.2
23	12,125	0	14.1	14.1
24	3358	8.5	16.0	18.2
25 (HQC)	30,518	0	14.1	14.1
26 (LQC)	1671	0	18.3	18.3
27 (NC) ^3^	28	0	17.4	17.4

^1^ Samples were either convalescent serum or serum from a Phase 1 clinical trial (excluding the NC, which was a pre-COVID-19 sample). ^2^ The overall assay precision is general assay precision, calculated from all 27 samples. ^3^ The NC GMC is much lower than LLoQ, sample acceptance criteria were not applied to obtain these values. Ab—antibody; EU—ELISA unit; GCV—geometric coefficient of variation; GMC—geometric mean concentration; HQC—high-quality control; LQC—low-quality control; NC—negative control; rS—recombinant spike protein.

**Table 2:** IgG assay ancestral strain assay specificity.

Assay Specificity						
Sample	Sample Source	Assay Buffer	Incubated with Homologous rS Protein, 4 µg/mL		Incubated with Homologous rS Protein, 2 µg/mL	
		Ab (EU/mL)	Ab (EU/mL)	% Inhibition	Ab (EU/mL)	% Inhibition
1	Phase 1 trial	16,077	877	94.5	1699	89.4
2	Phase 1 trial	1219	139	88.6	224	81.6
3	Phase 1 trial	131,959	6791	94.9	12,057	90.9
4	Phase 1 trial	35,640	2161	93.9	2446	93.1
5	Phase 1 trial	6353	632	90.1	1015	84.0
6	Phase 1 trial	574	67	88.3	94	83.6
**Antibody Cross-reactivity**						
MERS-CoV rS Protein						
**Sample**	**Sample Source**	**Assay Buffer**	**Incubated with** **MERS-CoV rS Protein, 4 µg/mL**		**Incubated with** **MERS-CoV rS Protein,** **2 µg/mL**	
		**Ab (EU/mL)**	**Ab (EU/mL)**	**% Inhibition**	**Ab (EU/mL)**	**% Inhibition**
1	Phase 1 trial	18,575	17,933	3.5	17,812	4.1
2	Phase 1 trial	1764	1448	17.9	1443	18.2
3	Phase 1 trial	132,825	109,291	17.7	110,509	16.8
4	Phase 1 trial	31,774	26,580	16.3	27,783	12.6
5	Phase 1 trial	7799	6404	17.9	6361	18.4
6	Phase 1 trial	646	484	25.1	586	9.3
**SARS-CoV-1 rS Protein**						
**Sample**	**Sample Source**	**Assay Buffer**	**Incubated with** **SARS-CoV-1** **rS Protein, 4 µg/mL**		**Incubated with** **SARS-CoV-1** **rS Protein, 2 µg/mL**	
		**Ab (EU/mL)**	**Ab (EU/mL)**	**% Inhibition**	**Ab (EU/mL)**	**% Inhibition**
1	Phase 1 trial	19,392	16,789	13.4	17,769	8.4
2	Phase 1 trial	1765	1512	14.3	1551	12.1
3	Phase 1 trial	130,229	100,597	22.8	127,364	2.2
4	Phase 1 trial	29,804	30,897	−3.7	28,603	4.0
5	Phase 1 trial	6384	5501	13.8	5290	17.1
6	Phase 1 trial	531	478	10.0	457	13.9
**Irrelevant Proteins (RSV F Protein, Influenza HA)**						
**Sample**	**Sample Source**	**Assay Buffer**	**Incubated with** **RSV F Protein, 4 µg/mL**		**Influenza A/Kansas/14/2017 VLP, 4 HA Units**	
		**Ab (EU/mL)**	**Ab (EU/mL)**	**% Inhibition**	**Ab (EU/mL)**	**% Inhibition**
1	Phase 1 trial	20,879	18,936	9.3	17,321	17.0
2	Phase 1 trial	1936	1680	13.2	1725	10.9
3	Phase 1 trial	153,044	120,742	21.1	133,650	12.7
4	Phase 1 trial	34,951	31,896	8.7	33,289	4.8
5	Phase 1 trial	7791	7035	9.7	6893	11.5
6	Phase 1 trial	671	626	6.7	645	3.9

Ab—antibody; EU—ELISA unit; HA—hemagglutinin; rS—recombinant spike protein; RSV—respiratory syncytial virus; VLP—virus-like particles.

**Table 3:** IgG assay ancestral strain heme and lipid matrix effects.

Heme Matrix Effects					
Sample	Control	50% Hemolyzed		25% Hemolyzed	
	Ab (EU/mL)	Ab (EU/mL)	% Recovery	Ab (EU/mL)	% Recovery
1	2931	2821	96.2	2954	100.8
2	31,541	30,494	96.7	33,161	105.1
3	4101	3969	96.8	4137	100.9
4	61,022	63,570	104.2	63,406	103.9
5	342	324	94.7	351	102.6
6	40 (<200)	33 (<200)	N/A	27 (<200)	N/A
**Lipid Matrix Effects**					
**Sample**	**Control**	**5.0 mg/mL** **Triglycerides**		**2.5 mg/mL** **Triglycerides**	
	**Ab (EU/mL)**	**Ab (EU/mL)**	**% Recovery**	**Ab (EU/mL)**	**% Recovery**
1	2931	3173	108.3	2748	93.8
2	31,541	32,109	101.8	29,970	95.0
3	4101	4260	103.9	4339	105.8
4	61,022	72,766	119.2	73,059	119.7
5	342	351	102.6	315	92.1
6	40 (<200)	25 (<200)	N/A	25 (<200)	N/A

Ab—antibody; EU—ELISA unit.

**Table 4:** IgG assay ancestral strain linearity.

Sample	Parameter	Estimate	95% LCL	95% UCL
1	Slope	1.034	1.022	1.046
	Intercept	−0.171	−0.220	−0.121
	Residual Variability (% GSD)	0.015 (3.4%)	N/A	
	R^2^	0.9998	N/A	
2	Slope	1.011	0.999	1.022
	Intercept	−0.046	−0.091	−0.001
	Residual Variability (% GSD)	0.016 (3.8%)	N/A	
	R^2^	0.9998	N/A	

GSD—geometric standard deviation; LCL—lower confidence limit; N/A—not applicable; UCL—upper confidence limit.

## Data Availability

Requests for the data presented in this study will be considered by the corresponding author. The data are not publicly available because of proprietary subject and sample information.

## Supplementary Materials

The following supporting information can be downloaded at: https://www.mdpi.com/article/10.3390/microorganisms11071789/s1, Table S1: IgG assay ancestral strain assay selectivity; Table S2: Assay specificity in RSV and influenza vaccinated participants; Table S3: IgG assay robustness—lower incubation time limit results; Table S4: IgG assay robustness—upper incubation time limit results; Table S5: IgG assay variation at increasing time after addition of stop solution; Table S6: IgG assay results for SARS-CoV-2 variant strains; Table S7: IgG assay conversion of reference units (EU/mL) to international units (ancestral strain) (BAU/mL).
