# Supplementary material for: A Severe Acute Respiratory Syndrome Coronavirus 2 Anti-Spike Immunoglobulin G Assay: A Robust Method for Evaluation of Vaccine Immunogenicity Using an Established Correlate of Protection
Source: Microorganisms. 2023 Jul 11;11(7):1789. doi: 10.3390/microorganisms11071789 (PMC10383018; doi:10.3390/microorganisms11071789)
Supplement: Supplementary file 1 [file microorganisms-11-01789-s001.zip › microorganisms-2457221-supplementary.pdf]

microorganisms

MDPI

Supplement: A Severe Acute Respiratory Syndrome Coronavirus 2 Anti-Spike Immunoglobulin G Assay: A Robust Method for Evaluation of Vaccine Immunogenicity Using an Established Correlate of Protection

Supp Table 1. IgG assay ancestral strain assay selectivity.

Sample	Anti-rS Protein IgG Level (EU/mL)	Comment
1	120	<LLoQ of 200 EU/mL
2	74	<LLoQ of 200 EU/mL
3	38	<LLoQ of 200 EU/mL
4	45	<LLoQ of 200 EU/mL
5	34	<LLoQ of 200 EU/mL
6	64	<LLoQ of 200 EU/mL
7	69	<LLoQ of 200 EU/mL
8	56	<LLoQ of 200 EU/mL
9	28	<LLoQ of 200 EU/mL
10	41	<LLoQ of 200 EU/mL
11	103	<LLoQ of 200 EU/mL
12	320	>LLoQ
13	31	<LLoQ of 200 EU/mL
14	22	<LLoQ of 200 EU/mL
15	91	<LLoQ of 200 EU/mL
16	110	<LLoQ of 200 EU/mL
17	29	<LLoQ of 200 EU/mL
18	136	<LLoQ of 200 EU/mL
19	125	<LLoQ of 200 EU/mL
20	422	>LLoQ
21	48	<LLoQ of 200 EU/mL
22	86	<LLoQ of 200 EU/mL
23	47	<LLoQ of 200 EU/mL
24	144	<LLoQ of 200 EU/mL
25	31	<LLoQ of 200 EU/mL
26	95	<LLoQ of 200 EU/mL
27	60	<LLoQ of 200 EU/mL
28	80	<LLoQ of 200 EU/mL
29	188	<LLoQ of 200 EU/mL
30	80	<LLoQ of 200 EU/mL
31	215	>LLoQ
32	47	<LLoQ of 200 EU/mL
33	47	<LLoQ of 200 EU/mL
34	69	<LLoQ of 200 EU/mL
35	154	<LLoQ of 200 EU/mL
36	104	<LLoQ of 200 EU/mL
37	250	>LLoQ
38	362	>LLoQ
39	113	<LLoQ of 200 EU/mL
40	32	<LLoQ of 200 EU/mL

Ab—antibody; EU—ELISA unit; IgG—immunoglobulin G; LLoQ— lower limit of quantitation; rS—recombinant spike protein.

Supp Table 2. Assay specificity in RSV and influenza vaccinated participants

RSV Vaccinated[a]

Participant #	Anti-RSV F Protein Antibody Unit			Tested in Anti-SARS-CoV-2 rS Protein ELISA		
	**Pre-RSV F Vaccination (Screening)**	**Post-RSV F Vaccination (Day 14)**	**Post/Pre Ratio**	**Pre-RSV F Vaccination (Screening)**	**Post-RSV F Vaccination (Day 14)**	**Post/Pre Ratio**
1	591	48,415	81.920	426	375	0.880
2	200	25,824	129.120	100	226	2.260
3	200	11,442	57.210	100	100	1.000
4	200	30,367	151.835	414	336	0.812
5	1120	21,309	19.026	100	100	1.000

Influenza Vaccinated

Participant #	HAI Titer (A/Kansas/14/2017) (H3N2)			Tested in Anti-SARS-CoV-2 rS Protein ELISA		
	**Pre-Flu Vaccination(Day 0)**	**Post-Flu Vaccination(Day 28)**	**Post/Pre Ratio**	**Pre-Flu Vaccination(Day 0)**	**Post-Flu Vaccination(Day 28)**	**Post/Pre Ratio**
1	80	1280	16.000	100	100	1.000
2	20	320	16.000	100	100	1.000
3	20	160	8.000	100	100	1.000
4	40	320	8.000	100	100	1.000
5	40	320	8.000	100	100	1.000

[a]RSV F ELISA LLOQ = 400 EU/mL, EU of 200 is used in calculation when < LLOQ; SARS-CoV-2 S protein ELISA LLOQ = 200 EU/mL, EU of 100 is used in calculation when < LLOQ.

[b]A/Kansas/14/2017 HAI titer LLOQ = 10; SARS-CoV-2 S protein ELISA LLOQ = 200 EU/mL, EU of 100 is used in calculation when < LLOQ.

ELISA—enzyme-linked immunosorbent assay; HAI—hemagglutination inhibition; RSV—respiratory syncytial virus; S—spike protein; SARS-CoV-2—severe acute respiratory syndrome coronavirus 2.

Supp Table 3. IgG assay robustness - lower incubation time limits results

Sample #	Reference Value (EU/mL)	Short Incubation (EU/mL)	% Recovery	% Difference
1	47,648	45,897	96.3	-3.7
2	16,537	14,741	89.1	-10.9
3	9303	8818	94.8	-5.2
4	202,618	199,948	98.7	-1.3
5	2146	2047	95.4	-4.6
6	4890	4967	101.6	1.6
7	1447	1449	100.1	0.1
8	105,191	119,513	113.6	13.6
9	510	512	100.3	0.3
10	1067	900	84.3	-15.7
11	4629	4661	100.7	0.7
12	824	716	86.9	-13.1
13	35,042	32,937	94.0	-6.0
14	106,859	102,366	95.8	-4.2
15	101,122	97,805	96.7	-3.3
16	26,750	27,046	101.1	1.1
17	12,125	12,615	104.0	4.0
18	3358	3396	101.1	1.1

EU—ELISA unit.

Supp Table 4. IgG assay robustness - upper incubation time limits results.

Sample #	Reference Value (EU/mL)	Long Incubation (EU/mL)	% Recovery	% Difference
1	47,648	53,224	111.7	11.7
2	16,537	18,330	110.8	10.8
3	9303	10,602	114.0	14.0
4	202,618	235,086	116.0	16.0
5	2146	2488	115.9	15.9
6	4890	6035	123.4	23.4
7	1447	1573	108.7	8.7
8	105,191	126,414	120.2	20.2
9	510	580	113.6	13.6
10	1067	980	91.8	-8.2
11	4629	5167	111.6	11.6
12	824	880	106.8	6.8
13	35,042	36,853	105.2	5.2
14	106,859	126,669	118.5	18.5
15	101,122	87,806	86.8	-13.2
16	26,750	31,304	117.0	17.0
17	12,125	13,932	114.9	14.9
18	3358	3617	107.7	7.7

EU—ELISA unit.

Supp Table 5. IgG assay variation at different time points after addition of stop solution.

Sample	Immediately	15 minutes		30 minutes	
	Ab (EU/mL)	Ab (EU/mL)	% Difference	Ab (EU/mL)	% Difference
HQC	31,829	32,361	1.7	32,485	2.1
LQC	1592	1606	0.9	1605	0.8
NC	29 (<200)	30 (<200)	N/A	28 (<200)	N/A
HQC	27,065	26,771	-1.1	26,977	-0.3
LQC	1465	1461	-0.3	1469	0.3
NC	29 (<200)	28 (<200)	N/A	29 (<200)	N/A
HQC	36,079	36,423	1.0	36,048	-0.1
LQC	1687	1718	1.8	1719	1.9
NC	35 (<200)	34 (<200)	N/A	35 (<200)	N/A

Ab—antibody; EU—ELISA unit; N/A—not applicable.

Supp Table 6. IgG assay results for SARS-CoV-2 variant strains.

Parameter	Beta	Delta	Omicron BA.1	Omicron BA.5	Omicron XBB.1.5
Precision (total, intra-assay and inter-assay)	<20% GCV in 18/20 (90%)	<20% GCV in 19/20 (95%)	<20% GCV for 20/21 (95.2%)	<20% GCV in 21/21 (100%)	<20% GCV in 21/21 (100%)
Linearity	R^2 = 0.9999 and 0.9996	R^2 = 0.9999 and 0.9998	R^2 = 0.986 and 0.966	R^2 = 0.9988 and 0.9991	R^2 = 0.9985 and 0.9991
LLoQ	200 EU/mL	200 EU/mL	200 EU/mL	200 EU/mL	200 EU/mL
ULoQ	490,731 EU/mL	501,789 EU/mL	391,124 EU/mL	≥ 990,591 EU/mL	682,680 EU/mL
Specificity					
Selectivity	36/40 (90%) < LLoQ	33/40 (82.5%) <LLoQ	31/40 (77.5%) <LLoQ Pre/post-vaccination with RSV F or nano Flu: No changes in anti-rS IgG levels (except 1/5 [20%] for RSV F)	34/40 (85%) < LLoQ	33/40 (82.5%) <LLoQ
Specific Inhibition	Beta: 8/8 (100%) reduced by >50% Ancestral: 8/8 (100%) reduced by >50% SARS-CoV S: 5/8 (62.5%) reduced <20% MERS-CoV S: 3/8 (37.5%) reduced RSV F: 3/8 (37.5%) reduced	Delta: 7/8 (87.5%) reduced by >50% Ancestral: 6/7 (85.7%) reduced by >50% Beta: 3/7 (42.9%) for low concentration and 6/7 (85.7%) for high concentration reduced by >50% SARS-CoV S: 2/7 (28.6%) reduced significantly	BA.1: 8/8 (100%) reduced by >50% Ancestral: 8/8 (100%) reduced by >50% Beta: 7/8 (87.5%) reduced by >50% Delta; 8/8 (100%) reduced by >50% RSV F: 2/8 (25%) reduced significantly Influenza HA: 1/8 (12.5%) reduced significantly	BA.5: 7/8 (87.5%) reduced by >50% BA.1: 6-7/8 (75-87.5%) reduced by >50% Ancestral: 6/8 (75%) reduced by >50% RSV F: 1/8 (12.5%) reduced significantly Ebola GP: 0/8 (0%) reduced significantly	XBB.1.5: 8/8 (100%) reduced by >75% BA.5: 8/8 (100%) reduced by >75% Ancestral: 8/8 (100%) reduced by >65% RSV F: 1/8 (12.5%) reduced significantly Ebola GP: 0/8 (0%) reduced significantly

	Influenza HA: 0/8 (0%) reduced significantly	MERS CoV S: 1/7 (14.3%) reduced significantly<br>RSV F: 0/7 (0%) reduced significantly<br>Influenza HA: 1/7 (14.3%) reduced significantly			
Plate coating robustness (72 hours)	-	-	17/21 (21%) within 80-120% of baseline values	17/21 (81%) within 80-120% of baseline values	21/21 (100%) within 80-120% of baseline values

Ab—antibody; EU—ELISA unit; GCV—geometric coefficient of variation; GP—glycoprotein; HA—hemagglutinin; IgG—immunoglobulin G; LLoQ—lower limit of quantitation; rS—recombinant spike protein; RSV F—respiratory syncytial virus F protein; ULoQ—upper limit of quantitation.

Supp Table 7. IgG assay conversion of reference units (EU/mL) to international standard units (ancestral strain) (BAU/mL).

**Sample**	**Run 1**			**Run 2**			**Run 3**		
	**BAU/mL**	**EU/mL**	**EU/BAU Ratio**	**BAU/mL**	**EU/mL**	**EU/BAU Ratio**	**BAU/mL**	**EU/mL**	**EU/BAU Ratio**
HQC	1145	27870	24.3	1195	26296	22.0	1622	31717	19.6
	1206	32151	26.7	1398	33418	23.9	1578	34834	22.1
LQC	71	1737	24.5	66	1412	21.4	84	1856	22.1
	69	1915	27.8	71	1825	25.7	85	2031	23.9
NC	<5	<200	N/A	<5	<200	N/A	<5	<200	N/A
	<5	<200	N/A	<5	<200	N/A	<5	<200	N/A
NIBSC 20/150	1004	22621	22.5	977	22364	22.9	1258	24945	19.8
	1145	27430	24.0	994	20762	20.9	1251	26935	21.5
NIBSC 20/148	288	6837	23.7	310	6549	21.1	351	6769	19.3
	342	7951	23.2	287	6478	22.6	312	6972	22.3
NIBSC 20/144	113	2484	22.0	111	2366	21.3	129	2514	19.5
	113	2561	22.7	100	2164	21.6	123	2629	21.4
NIBSC 20/140	65	1514	23.3	65	1422	21.9	83	1669	20.1
	68	1553	22.8	53	1271	24.0	82	1720	21.0
NIBSC 20/142	<5	<200	N/A	<5	<200	N/A	<5	<200	N/A
	<5	<200	N/A	<5	<200	N/A	<5	<200	N/A
GMR			23.9			22.4			21.0
Overall GMR	22								

Ab—antibody; BAU—binding antibody unit; EU—ELISA unit; GMR—geometric mean ratio; HQC—high quality control; LQC—low quality control; N/A—not applicable; NC—negative quality control; NIBSC—National Institute for Biological Standards and Contro
